# 

**Published:** 1887-12

**Authors:** 


PROF. LOISETTE'S MEMORY DISCOVERY.—Prof. Loisette's new system of memory training; taught by correspondence at 237 Fifth Avenue, New York, seems to supply a general want. He has had two classes at Yale of 200 each, 250 at Meriden, 300 at Norwich, 100 Columbia law students, 400 at Wellesley College, and 400 at University of Pennsylvania, etc. Such patronage and the endorsement of such men as Mark Twain, Dr. Buckley, Prof. Wm. R. Harper, of Yale, etc., place the claim of Prof. Loisette upon the highest ground.

